# Age at first childbirth and breast cancer survival: a prospective cohort study

**DOI:** 10.1186/s13104-019-4864-1

**Published:** 2020-01-06

**Authors:** Johanna Aurin, Henrik Thorlacius, Salma Tunå Butt

**Affiliations:** Institution of Clinical Sciences Malmö, Department of Surgery, Skåne University Hospital Malmo, Lund University, 205 02 Malmö, Sweden

**Keywords:** Breast cancer risk, Age at first childbirth, Nulliparity, Survival, Reproductive factors

## Abstract

**Objective:**

Late age at first childbirth is a well-established risk factor for breast cancer. Previous studies have, however, shown conflicting results to whether late age at first childbirth also influences the prognosis of breast cancer survival. The aim of this study was to examine age at first birth in relation to survival after breast cancer diagnosis.

**Results:**

We used information from the Malmö Diet and Cancer study. At baseline 17,035 women were included. All women were followed from the year they developed breast cancer until they either died or until the end of follow-up. All women were asked how many children they had given birth to and were then divided into different groups, ≤ 20, > 20 to  ≤ 25, > 25 to  ≤ 30 and > 30. Nulliparous women form a separate group. Survival analyses were then performed using Cox proportional hazard survival analysis. Women in all age groups had a lower risk of breast cancer specific death as compared to the reference group ≤ 20, however non-significantly. Nulliparous women had a higher risk of breast cancer specific death as compared to the same reference group, however these results were not statistically significant. We could not see any negative effect of late first childbirth on breast cancer specific survival.

## Introduction

Late age at first childbirth (> 30 years old) has shown to be a risk factor for breast cancer, [1, 2] however previous studies have shown conflicting results whether age at first birth also influences the prognosis after breast cancer. Some studies show no association between age at first childbirth (AFB) and breast cancer specific [3] or overall survival [3–7]. Other studies suggest that an older age at first childbirth may lead to a poorer [8] or better breast cancer specific [9–11] or overall survival [12, 13]. A study conducted in Sweden was able to show a connection between late childbirth and more aggressive breast cancer subgroups [14]. However, that study did not investigate weather that also translated into a poorer breast cancer specific survival. The age at first childbirth has increased among Swedish mothers since 1970. In 1970 the mean age at first childbirth was 24 years old, in 2016 the mean age at first childbirth had increased to 29 years old [15]. If late age at first childbirth does lead to poorer breast cancer survival, this is an important factor to consider for future childbearing practices.

The aim of this study was to examine age at first childbirth in relation to breast cancer survival among women diagnosed with breast cancer.

## Main text

### Materials and methods

#### MDCS Malmö Diet and cancer study

All female residents in Malmö, Sweden, who were born between 1923 and 1950, were between the years 1991 and 1996 asked to participate in a population based prospective cohort study, the Malmö Diet and Cancer Study (MDCS). MDCS was initiated to study the association of dietary factors with different cancers but was later on expanded to study different lifestyle factors with cancer. A total of 40% of the invited women accepted the invitation and 17,035 women participated in the baseline examination. At inclusion the participants accomplished a dietary assessment, a self-administrated questionnaire and anthropometric body measurement. A trained nurse measured body weight and height at the baseline inclusion. The questionnaire provided information such as parity, age at first birth, age at menarche and year of menopause, occupation, smoking and alcohol habits [16].

#### Study population

At baseline examination 476 women had already been diagnosed with breast cancer and these women were excluded from the study.

The patients were followed from the year they were diagnosed with breast cancer until they either died or until the end of follow up in 31 December 2011. A total of 1498 women were diagnosed with invasive breast cancer and out of these 246 did not have information on tumour characteristics and 11 did not have valid follow-up data. Hence the study population for this study consisted of 765 women.

#### Scoring and classification

Age at first childbirth was retrieved from questionnaire information. All women were asked how many children they had given birth to and in what years and age at first childbirth was then calculated. The variable was then divided in four age groups, ≤ 20, > 20– ≤ 25, > 25– ≤ 30 and > 30. Nulliparous women form a separate group.

#### Follow-up

The end of follow up was set to 31 December 2011. There was a linkage between the MDCS and the Swedish Cancer Registry. Tumour end points were retrieved from this register (until December 31 2007). Information from the Southern Swedish Regional Tumour Registry was also retrieved due to a delay of registration to the national registry.

There was also a linkage to the Swedish Cause of Death registry and vital statuses of all patients were retrieved from this register by December 31 2011.

#### Statistical analyses

All patients in the study were divided into different categories depending on what age they gave birth to their first child. The different categories were then compared regarding established risk factors for breast cancer. Statistical analyses were performed using Cox proportional hazard survival analysis yielding hazard ratios with 95% confidence interval. Women who had given birth to their first child before the age of 20 years were used as a reference group.

We adjusted for lifestyle factors associated with breast cancer risk i.e. socioeconomic status, alcohol consumption, age at baseline, age at menarche, age at menopause, BMI, parity and smoking. We also adjusted for all available breast cancer characteristics; tumor size, lymph node status, grade, ER, HER2, PgR, Ki67, Cyclin D1 and p27.

All confounders were tested one by one in relation to survival in order to see which confounder affected the results the most.

SPSS 25 was used to perform all statistical analyses.

### Results

From inclusion until the end of follow-up a total number of 765 women had been diagnosed with incident breast cancer (including those diagnosed with carcinoma in situ and bilateral breast cancer). By the end of follow up 115 women had died of breast cancer.

#### Distribution of risk factors of breast cancer in different ages at first childbirth

Women younger than 20 years at first childbirth were more likely to work with manual labour compared to nulliparous and to women who were older when they first gave birth. Nulliparous and women in the age group 26– ≤ 30 were more likely to never have been smokers. Those who were younger than 25 years old when they first gave birth were more likely to be current smokers and more likely to be pre/perimenopausal compared to all other age groups. The younger the age at first childbirth the more children the women were likely to have given birth to (Table 1). All other potential confounders were evenly distributed throughout all other age groups.

**Table 1:** Distribution of risk factors in different ages at first childbirth

Factor	Nullipara (*n *= 83)	< 20 years (*n *= 114)	21 to ≤ 25 years (*n *= 252)	26 to ≤ 30 years (*n *= 188)	> 30 years (*n *= 74)	Missing (*n *= 54)
Age at baseline, year (765) Mean (SD)	*57.3 (7.7)*	*55.5 (6.3)*	*56.5 (7.1)*	*57.2 (7.1)*	*56.8 (7.9)*	*57.1 (5.3)*
Socioeconomical status (720)						
Manual worker	26.5	49	39.4	25.7	26.4	11.1
Nonmanual worker	68.7	47	55.3	68.4	65.3	83.3
Employer-self-employed	4.8	4	5.3	5.9	8.3	5.6
Body mass index (765) Mean (SD)	*25.6 (4.1)*	*26.6 (4.1)*	*25.5 (4.1)*	*25.1 (3.4)*	*25.6 (4.2)*	*23.9 (3.2)*
Alcohol consumption (765)						
Nothing last year	8.4	11.4	10.7	10.1	9.5	0
Something last year (not last month)	9.6	13.2	10.7	6.9	16.2	1.3
Something last month	81.9	74.6	78.2	83	74.3	94.4
Smoking (765)						
Never	50.6	35.1	40.9	51.6	43.2	33.3
Current	26.5	31.6	30.6	20.2	25.7	38.9
Ex	22.9	33.3	28.6	28.2	31.1	27.8
Age at menarche (722)						
≤ 12	21.7	26.3	24	14.2	21.6	16.7
> 12 to < 15	47	53.5	48.4	59	56.8	50
≥ 15	31.3	20.2	27.6	26.8	21.6	33.3
Age at menopause (714)						
Pre/perimenopausal	31.7	38.9	38	32.2	34.2	27.8
≤ 45	12.2	14.2	12.2	8.2	13.7	16.7
> 45 to < 53	43.9	34.5	33.9	46.4	32.9	44.4
≥ 53	12.2	12.4	15.9	13.1	19.2	11.1
Parity (711)						
Nullipara	100	0	0	0	0	0
1	0	19.3	11.1	25	63.5	0
2	0	38.6	58.7	58	35.1	–
3	0	27.2	24.2	14.9	1.4	0
≥ 4	0	14.9	6	2.1	0	0

Column percent (mean and SD in italics)

#### Age at first childbirth in relation to risk of death after breast cancer diagnosis

Nulliparous women had a poorer breast cancer specific survival as compared to women who were < 20 years of age at AFB, however, these results did not reach statistical significance (Table 2). Women of all other age categories had a better breast cancer specific survival as compared to those who were ≤ 20 years AFB (Table 2), but these results were not statistically significant either.

**Table 2:** Risk of death in breast cancer

Factor	Number of cases	Unadjusted HR	Number of cases	Adjusted HR^a^	Number of cases	Adjusted HR^b^
Age at first birth	765	–	685	–	382	–
Nullipara	83	1.33 (0.69–2.53)	82	1.58 (0.51–4.89)	50	1.42 (0.16–12.26)
< 20	114	1.00	113	1.00	72	1.00
21 to ≤ 25	252	0.76 (0.43–1.33)	240	0.64 (0.35–1.15)	120	0.58 (0.26–1.32)
26 to ≤ 30	188	0.70 (0.38–1.28)	179	0.67 (0.35–1.27)	96	0.88 (0.38–2.02)
> 30	74	0.69 (0.31–1.53)	71	0.80 (0.33–1.90)	36	0.59 (0.19–1.81)
Missing	54	1.27 (0.60–2.68)	18	1.43 (0.34–5.97)	8	0.49 (0.34–7.38)

^a^95% CI for HR. Adjusted for socioeconomic status, alcohol consumption, age at baseline, age at menarche, age at menopause, BMI, parity and smoking

^b^95% CI for HR. Adjusted for all the factors stated above and for tumor size, lymph node status, grade, ER, HER2, PgR, Ki67, Cyclin D1 and p27

In this study we investigated the connection between survival after breast cancer diagnosis and AFB. We found no effect of late first childbirth on breast cancer specific survival.

### Discussion

#### Methods and material of this study

The participants in the MDCS have shown to have a higher incidence of breast cancer and are possibly selected towards higher social groups compared to the rest of the population in Malmö. An earlier study of the MDCS has shown that the participating women are healthier that the general population in Malmö [17].There prevalence of obesity and smoking is the same in the study as in the rest of the population in Malmö. There is no information about other factors that could influence incidence or survival outside the study group. It has been considered that the higher incidence of breast cancer among the studied population could be explained by a higher participation in mammography screening in the studied group compared to the rest of the population [16, 17]. The higher incidence of breast cancer in the study group is not considered to affect our results since the aim was to study survival after breast cancer diagnoses in relation to age at first birth. By the end of follow up 115 women had died of breast cancer, i.e. 15%. As previously described the women in MDCS are healthier than the background population [17]. The survival rate could, however, be affected by the fact that the women in the study generally are more educated compared to the rest of the population. Since the results are adjusted for various demographic factors and socioeconomic status we do consider it to be possible to obtain valid results.

#### Previous studies

In accordance to some previous studies our results indicate that an older age at first childbirth leads to a better prognosis after breast cancer diagnosis [9–13]. Contrary to our findings, some other studies have reported that an older age at first childbirth may lead to a poorer survival [8] or that there is no connection between AFB and survival [3–7].

Our results indicate that nulliparity potentially leads to a poorer prognosis. Earlier studies have found a connection between nulliparous women with premenopausal breast cancer and better survival compared to parous women [5, 10]. Among postmenopausal breast cancer nulliparity has shown to be a risk factor for a poorer outcome after diagnosis [10, 18]. Previous childbirth does not seem to affect the chances of survival when breast cancer of all age groups is studied [5, 9, 19].

#### Potential explanations

Women who were young at AFB were more likely to have a lower socioeconomic status compared to women who were older when they first gave birth. Low socioeconomic status is associated with factors that are prognostically negative for survival such as late detection of the tumour and poorer compliance to treatment [5, 20], which could be one possible explanation to the indication towards a poorer survival among women with early AFB.

One earlier study has shown that nulliparity leads to breast cancer with more aggressive subtypes [14], which could be a possible explanation to the poorer prognosis among these women. In earlier studies it has been suggested that pregnancy induces an adverse effect, which leads to a poorer prognosis in breast cancer during and the years after the pregnancy, this effect does however seem to be transient and postmenopausal parous women have a better prognosis compared to nulliparous. In our study approximately two-thirds of the women were postmenopausal with a slightly higher ratio among the nulliparous compared to the parous, which supports this hypothesis.

### Conclusion

In this study we could not find any association between age at first childbirth and breast cancer specific survival.

## Limitations

Information on recurrence of breast cancer had been of great interest in stratifying our data on mortality, however this data was not available.

## Data Availability

Data from Malmoe Diet And Cancer Study can be attained by filling in application to the MDCS committee. All data is confidential regarding patient information. https://www.malmo-kohorter.lu.se/malmo-kost-cancer-mkc

## Abbreviations

AFB: age at first childbirth
MDCS: Malmö Diet and Cancer Study

## References

[1] Kelsey JL, Gammon MD (1991). The epidemiology of breast cancer. CA Cancer J Clin..

[2] Albrektsen G, Heuch I, Hansen S, Kvale G (2005). Breast cancer risk by age at birth, time since birth and time intervals between births: exploring interaction effects. Br J Cancer.

[3] Warren Andersen S, Newcomb PA, Hampton JM, Titus-Ernstoff L, Egan KM, Trentham-Dietz A (2011). Reproductive factors and histologic subtype in relation to mortality after a breast cancer diagnosis. Breast Cancer Res Treat.

[4] Barnett GC, Shah M, Redman K, Easton DF, Ponder BA, Pharoah PD (2008). Risk factors for the incidence of breast cancer: do they affect survival from the disease?. J Clin Oncol.

[5] Reeves GK, Patterson J, Vessey MP, Yeates D, Jones L (2000). Hormonal and other factors in relation to survival among breast cancer patients. Int J Cancer.

[6] Morrison AS, Lowe CR, MacMachon B, Ravnihar B, Yuasa S (1977). Incidence risk factors survival in breast cancer: report on five years of follow-up observation. Eur J Cancer.

[7] Lees AW, Jenkins HJ, May CL, Cherian G, Lam EW, Hanson J (1989). Risk factors and 10-year breast cancer survival in northern Alberta. Breast Cancer Res Treat.

[8] Alsaker MD, Opdahl S, Asvold BO, Romundstad PR, Vatten LJ (2011). The association of reproductive factors and breastfeeding with long term survival from breast cancer. Breast Cancer Res Treat.

[9] Kroman N, Wohlfahrt J, Andersen KW, Mouridsen HT, Westergaard T, Melbye M (1998). Parity, age at first childbirth and the prognosis of primary breast cancer. Br J Cancer.

[10] Rosenberg L, Thalib L, Adami HO, Hall P (2004). Childbirth and breast cancer prognosis. Int J Cancer.

[11] Schouten LJ, Hupperets PS, Jager JJ, Volovics L, Wils JA, Verbeek AL, Blijham GH (1997). Prognostic significance of etiological risk factors in early breast cancer. Breast Cancer Res Treat.

[12] Greenberg ER, Vessey MP, McPherson K, Doll R, Yeates D (1985). Body size and survival in premenopausal breast cancer. Br J Cancer.

[13] Trivers KF, Gammon MD, Abrahamson PE, Lund MJ, Flagg EW, Kaufman JS, Moorman PG, Cai J, Olshan AF, Porter PL, Brinton LA, Eley JW, Coates RJ (2007). Association between reproductive factors and breast cancer survival in younger women. Breast Cancer Res Treat.

[14] Butt S, Borgquist S, Anagnostaki L, Landberg G, Manjer J (2009). Parity and age at first childbirth in relation to the risk of different breast cancer subgroups. Int J Cancer.

[15] SCB SS (2017). Birth intervals—how long do parents wait before they have a second child?.

[16] Manjer J, Carlsson S, Elmstahl S, Gullberg B, Janzon L, Lindstrom M, Mattisson I, Berglund G (2001). The Malmo Diet and Cancer Study: representativity, cancer incidence and mortality in participants and non-participants. Eur J Cancer Prev.

[17] Manjer J, Elmstahl S, Janzon L, Berglund G (2002). Invitation to a population-based cohort study: differences between subjects recruited using various strategies. Scand J Public Health.

[18] Butt S, Borgquist S, Garne JP, Landberg G, Tengrup I, Olsson A, Manjer J (2009). Parity in relation to survival following breast cancer. Eur J Surg Oncol.

[19] Ewertz M, Gillanders S, Meyer L, Zedeler K (1991). Survival of breast cancer patients in relation to factors which affect the risk of developing breast cancer. Intern J Cancer.

[20] Karjalainen S, Pukkala E (1990). Social class as a prognostic factor in breast cancer survival. Cancer.

